# Supression of chronic central pain by superoxide dismutase in rats with spinal cord injury: Inhibition of the NMDA receptor implicated

**DOI:** 10.3892/etm.2014.1878

**Published:** 2014-08-04

**Authors:** YONG-GANG XIE, HONG-JIE MU, ZHEN LI, JIA-HAI MA, YUE-LAN WANG

**Affiliations:** 1 Department of Anesthesiology, Qianfoshan Hospital, Affiliated to Shandong University Medical College, Jinan, Shandong 250014, P.R. China; 2 Department of Anesthesiology, Yuhuangding Hospital Affiliated to Qingdao University Medical College, Yantai, Shandong 264000, P.R. China; 3 Department of Orthopedics, Yantaishan Hospital Affiliated to Medical College of Taishan, Yantai, Shandong 264000, P.R. China; 4 Department of Otorhinolaryngology, Yantaishan Hospital Affiliated to Medical College of Taishan, Yantai, Shandong 264000, P.R. China

**Keywords:** chronic central pain, phosphorylated N-methyl-D-aspartate receptor subunit 1, superoxide dismutase, spinal cord injury, inhibition

## Abstract

Superoxide dismutase (SOD) is used to manage chronic pain, including neuropathic and inflammatory pain. However, data regarding the clinical effectiveness are conflicting and the neurophysiological mechanism of SOD has yet to be elucidated. The aim of the present study was to investigate whether SOD relieved chronic central pain (CCP) following spinal cord injury (SCI) and the possible underlying mechanisms. A CCP model was established using the Allen method and the CCP of the rats was measured using the paw withdrawal threshold. SOD was administered intraperitoneally following the establishment of CCP as a result of SCI. The results demonstrated that SOD relieved CCP in rats following SCI. In addition, the expression of spinal phosphorylated N-methyl-D-aspartate(NMDA) receptor subunit 1 (pNR-1) was inhibited in the CCP rats that had been treated with SOD. These observations indicated that SOD reduced mechanical allodynia and attenuated the enhancement of spinal pNR1 expression in rats with CCP. In addition, the results indicated that superoxide, produced via xanthine oxidase, and the participation of superoxide and nitric oxide (NO) as a precursor of peroxynitrite in NMDA, were involved in the mediation of central sensitization. Therefore, the observations support the hypothesis that SOD may have a potential therapeutic role for the treatment of CCP following SCI via the manipulation of superoxide and NO.

## Introduction

Chronic central pain (CCP) often occurs as a result of spinal cord injury (SCI). It is characterized by spontaneous pain, hyperalgesia (increased pain due to painful stimuli) and allodynia (pain due to normally non-painful stimuli). Due to the long-duration, slow recovery rate and difficult management, CCP is regarded as one of the most obstinate pain syndromes and a major challenge for patients following SCI (1).

An important consideration in the study of CCP following SCI using an animal model is the pathological and/or behavioral responses associated with human SCI. Numerous experimental models, including photochemical, hemisection and clip compression models, have been developed to replicate the complex pathophysiological mechanisms of SCI (2–4). Although these models share a number of pathological characteristics with the human condition, the specific neural substrates responsible for the injury-induced abnormal sensations have yet to be elucidated. Yezierski *et al* demonstrated that elevated excitatory amino acid levels were associated with SCI (5). In addition, injection of N-methyl-D-aspartate (NMDA) into the ventral horn of the spinal cord has been found to induce marked neuronal degeneration and acute inflammation in the grey and white matter (6). Previous studies have also demonstrated that reactive oxygen species (ROS) are involved in persistent pain, including neuropathic and inflammatory pain. Coderre *et al* (7,8) reported that ROS are correlated with the generation of allodynia. Using a peripheral nerve injury (spinal nerve ligation) model, free radical scavengers, including phenyl-N-t-butylnitrone (PBN) and 5,5-dimethyl-1-pyrroline-N-oxide, were found to decrease mechanical allodynia (9). In addition, systemically or intrathecally injected antioxidants were shown to reduce the formalin-induced nociceptive response in the hindpaws of mice (10). Furthermore, following peripheral nerve injury, such as sciatic nerve transection, ROS generation in the spinal cord was found to increase, while superoxide dismutase (SOD) activity decreased, and the mitochondrial ROS inside the spinal dorsal horn neurons in the spinal nerve ligation model was found to decrease (11,12).

Using a neuropathic pain model, the enhancement of NMDA receptor phosphorylation by ROS has become increasingly studied as an important mechanism of central sensitization (13,14). From these observations, it has been hypothesized that the development of the allodynia mechanism may result in an increase in ROS in the spinal cord and NMDA receptor phosphorylation may induce central sensitization.

Therefore, in the present study, animal experiments were performed to investigate whether NMDA receptor phosphorylation is involved in the central sensitization of CCP rats following SCI. In addition, SOD was administered to investigate whether the enzyme inhibits the phosphorylation of NMDA receptors and alleviates CCP following SCI.

## Materials and methods

### Animals

A total of 30 SPF grade Male Sprague-Dawley rats (weight, 230–250 g) were provided by the Institute of Animal Research of the Chinese Academy of Science (Beijing, China). The experimental protocols were approved by the Animal Use and Care Committee of Shandong University Health Science Center (Jinan, China).

### CCP model

Adult Sprague-Dawley rats were spinally contused at L1, as previously described by Yezierski *et al* (15) and Allen *et al* (16). Briefly, under 10% chlorohydrate anesthesia (3 ml/kg body weight, i.p.), the surgical field was shaved and a longitudinal incision was made that exposed several segments. A laminectomy was then performed at the two vertebral segments (T13-L2). The spinal cord was contused using a 20-g copper bar falling from 20 cm. The muscle and skin were then sutured and the wound was treated with penicillin.

### Pain behavior experiments

Behavioral tests were blindly performed by an examiner, who was provided no information concerning the experimental treatment. In order to observe the behaviors, the rats were placed in a transparent acryl box installed on a wire net. After a 15-min adaptation period, mechanical allodynia was observed.

A Dynamic Plantar Aesthesiometer (Ugo Basile, Comeril, Italy), operated using an automated Von Frey’s method, was used for the measurement of mechanical allodynia. Once the animals had adapted to the wire net, a straight metal Von Frey filament (diameter, 0.5 mm) was placed at the plantar surface of the ipsilateral hindpaw and the force (maximum force, 50 g) was increased gradually until a withdrawal response was observed, in order to measure the force required. This was repeated four times with a minimum interval of 10 sec to measure the paw withdrawal threshold. Mechanical allodynia was examined and the average threshold was calculated for the contralateral hindpaw in the same manner. The measurement data prior to SCI was set as the baseline value and the ipsilateral and contralateral data were measured on day 14 following SCI, when mechanical allodynia was the highest.

### Immunohistochemistry

Rats were anesthetized using 10% chlorohydrate (4.5 ml/kg body weight, i.p.; Yangzhou Aoxin Regent Factory of China, Yangzhou, China). The rats were then perfused transcardially with 150–200 ml normal saline followed by 200 ml fixative, consisting of 4% paraformaldehyde in 0.1 M phosphate buffered saline (PBS; pH 7.4). The L1 spinal segment was removed, postfixed in 4% paraformaldehyde in PBS for 4–6 h, and then transferred to 20% sucrose for 24–48 h. Transverse sections of 12-μm thickness were cut using a cryostat. One out of every 5–6 sections through the L1 spinal segment was collected and mounted on gelatin-subbed slides for immunohistochemical labeling for the phosphorylated NMDA receptor subunit 1 (pNR1). A total of six sections from each rat were collected, with 10 rats in each group.

The sections were stained immunohistochemically for pNR1 using the Avidin Biotin Complex method (Hebei Yongchuan Chemical Industry of China, Hebei, China). To reduce the possibility of diaminobenzidine tetrahydrochloride (DAB) reacting with endogenous peroxidases in the red blood cells in the tissues, and additionally to increase the penetration of the antibody into the tissue, the tissue sections were rinsed in 0.3% H_2_O_2_ methanol solution for 30 min prior to incubation with pNR1 antisera (Upstate Biotechnology, Lake Placid, NY, USA). The tissue sections were then incubated sequentially with rabbit anti-pNR1 (1:1,600; Upstate Biotechnology, Lake Placid, NY, USA) overnight at 4°C, biotinylated goat anti-rabbit immunoglobulin G for 1 h at room temperature and avidin-biotin-peroxidase reagent using a Histostain™-SP kit (SP-9001-3; Zymed Laboratories, Inc., South San Francisco, CA, USA) for 30 min at room temperature. All incubation steps were preceded by three rinses in PBS for 5 min. The tissue samples was immunoreacted for pNR1 in 0.05% DAB and 0.03% H_2_O_2_ in 0.01 M PBS for 2–3 min to yield a brown reaction product. The DAB step was preceded and followed by three rinses with 0.01 M PBS for 5 min. The sections were dehydrated in a series of dilutions of ethanol in water and xylene, and the slides were mounted. To confirm the specificity of the immunolabeling, control slides were exposed to diluted normal goat serum (manufactured in New Zealand). Control slides that omitted the primary antibody were consistently negative. The specificity of the antisera was tested in a previous study (17).

### Statistical analysis

Data were analyzed using SPSS 12.0 software (SPSS, Inc., Chicago, IL, USA) and results are presented as the mean ± standard error of the mean. Comparisons between the mean values of groups were analyzed using t-tests, and one-way or two-way analysis of variance where appropriate, followed by the Students Newman-Keul’s test. P<0.05 was considered to indicate a statistically significant difference.

## Results

### Effect of SOD on rats with CCP

**Figure 1:**
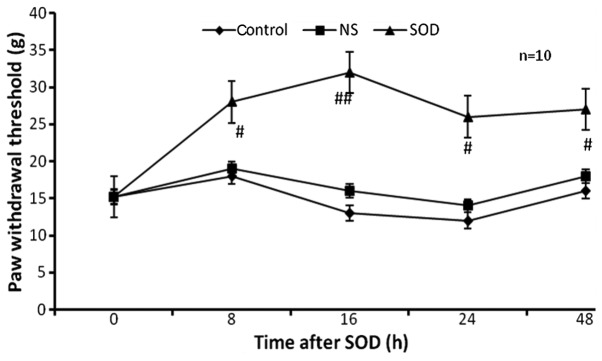
Inhibitory effect of SOD on rats with CCP (time-effect curve). Vertical bars represent the standard error of the mean, and differences between the groups were analyzed using two-way analysis of variance, followed by the Students Newman-Keul’s test. ^#^P<0.05 and ^##^P<0.01, vs. pre-injection of SOD in the same group. SOD, superoxide dismutase; CCP, chronic central pain.

Behavioral tests were performed at day 14 following SCI. Rats with SCI that had a paw withdrawal threshold of <20 g were considered to have CCP. The CCP rats were randomly assigned into three groups, with 10 rats in each group. Intraperitoneal injection of 4,000 U/kg SOD (SOD group; n=10) and 1 ml normal saline (NS group; n=10) were administered. SOD was obtained from Sigma-Aldrich (St. Louis, MO, USA) and was resolved in NS prior to the injection. In addition, 10 rats with CCP received no treatment as a control group. The dose of NS and SOD were based on previous conventional studies associated with neuropathic pain models (17,18). The paw withdrawal threshold was observed at 8, 16, 24 and 48 h following injection with SOD (Fig. 1). In the control and NS groups, the paw withdrawal threshold was stable over a period of 48 h. By contrast, in the SOD group, the paw withdrawal threshold increased markedly between a basal level of 15.2±1.8 and 30.4±1.6 g (P<0.01) at 16 h following the injection of SOD, and remained high for 8, 24 and 48 h (P<0.05).

### Effect of repeated injections of SOD on rats with CCP

**Figure 2:**
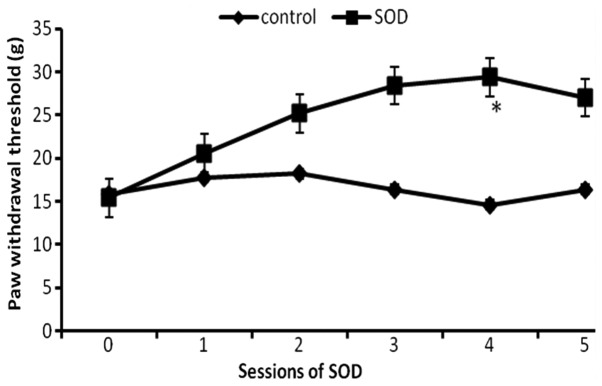
Analgesic effects of repeated treatment with SOD on CCP rats. The paw withdrawal threshold was increased following injection with SOD as compared with the control group (^*^P<0.05). SOD, superoxide dismutase; CCP, chronic central pain.

Since the aforementioned results demonstrated that SOD inhibited CCP, it was then investigated whether SOD has a cumulative analgesic effect with multiple treatments. The CCP rats were divided into two groups (n=12) at day 16 following SCI. The control group were restrained in the holder, while the SOD group were administered SOD intraperitoneally every two days for five sessions. The paw withdrawal threshold was assessed prior to each administration of SOD (Fig. 2). At the baseline, the paw withdrawal thresholds for the two groups were almost identical; however, the withdrawal thresholds progressively increased in the SOD group as compared with the control group (P<0.05), indicating that the degree of CCP decreased with repeated treatments of SOD.

### Measurement of pNR1

**Figure 3:**
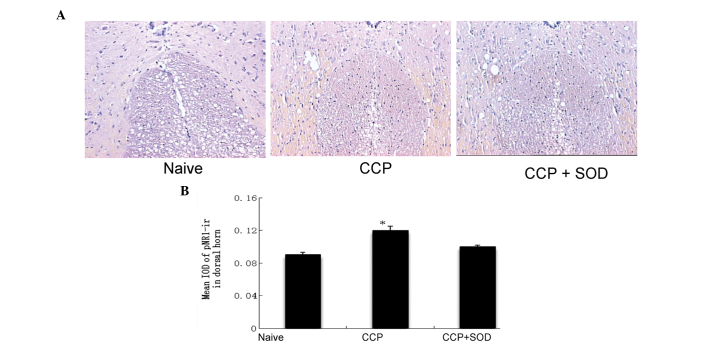
(A) Immunostaining of pNR1 in sections of the dorsal horn in the L1 spinal cord segments of rats. (B) Statistical analysis of the mean IOD of pNR1 immunostaining. Rats were divided into three groups: Naive, CCP and CCP + SOD. ^*^P<0.01, vs. naive group; ^*^P<0.05, vs. CCP + SOD group (one-way analysis of variance). pNR1, phosphorylated N-methyl-D-aspartate receptor subunit 1; IOD, integrated optical density; CCP, chronic central pain; SOD, superoxide dismutase. Magnification, ×2.

Following five sessions of SOD injections, the expression levels of pNR1 were analyzed in the L1 spinal cord of rats in the SOD, control and naive groups. The naive group had no intervention. pNR1 immunoreactivity was enhanced significantly in the superficial laminae of the spinal cord dorsal horn in the control CCP rats when compared with the naive rats. However, no statistically significant difference was observed between the SOD and naive groups (Fig. 3A). Histological images were further analyzed densitometrically using an HMIAS-2000 Microimaging Collection and Analysis System and the mean integrated optical density values of pNR1 in the superficial laminae (I–II) were measured (Fig. 3B). The increased expression of pNR1 immunoreactivity in the CCP rats was significantly suppressed by treatment with SOD.

## Discussion

In the present study, a long-lasting reduction in CCP was observed following the injection of SOD. In addition, SOD was shown to significantly decrease the expression of pNR1 in sections of the dorsal horn. These observations indicate that superoxide, mediated by the inflammatory reaction following SCI, may cause hyperalgesia by inducing NMDA receptor phosphorylation in spinal cord cells. Furthermore, SOD may decrease the phosphorylation of the NMDA receptor, inhibiting the generation of superoxide and reducing CCP.

ROS are involved in intracellular signaling transduction pathways as the normal derivatives of oxygen metabolism. ROS are balanced by the antioxidant capacity of the body; however, when the balance is lost, ROS increase and cause oxidative stress. Superoxide functions as a proinflammatory factor by increasing endothelial permeability, generating chemotactic factors, such as leukotriene B4, and inducing neutrophils. Superoxide reacts with nitric oxide (NO) to form peroxynitrite, which is involved in post-ischemia cellular injury by continuously generating superoxide and inactivating intrinsic manganese SOD (MnSOD) by nitration (20,21). The pain-associated role of ROS was further investigated by Kim *et al* (22), who demonstrated that plasma superoxide was inhibited by high levels of allopurinol prior to ischemia in an SCI model (22). These observations indicated that ROS are associated with an increase in nociceptor sensitivity, as well as the pain transmission pathway and mechanism, and may be involved in the chronic pain of peripheral tissues. The development and maintenance of chronic pain is known to be associated with central sensitization. Previous studies have focused on superoxide and NO as the ROS predominantly involved in central sensitization, as well as the role of superoxide in association with mitochondrial MnSOD (12,13). Park *et al* (12) demonstrated that mitochondrial ROS increased in the spinal dorsal horn of a spinal nerve ligation model. In addition, intraplantar injection of carrageenan in rats caused hyperalgesia, and increased MnSOD nitration in the spinal cord was found to decrease hyperalgesia. Following peripheral nerve injury, the release of excitatory amino acids, including glutamate, in the dorsal horn stimulates the NMDA receptor, resulting in the production of superoxide and NO via the activation of enzymatic cascades through the NMDA receptor. Peroxinitrate, formed as a result of superoxide and NO, causes MnSOD nitration, resulting in spinal cord hypersensitivity and an increase in central sensitization mediated by NMDA. (13,23).

Physiologically, spinal cord central sensitization is defined as the increased reactivity of the spinal dorsal horn to peripheral nociceptor stimuli. The induction of spinal cord central sensitization is associated with NMDA receptor subtypes, which have previously been categorized. The NMDA receptor is activated via the phosphorylation of NR1 (24). The decrease in pNR1 immunoreactive neurons in the spinal dorsal horn is consistent with the decrease in hyperalgesia following the injection of PBN (9), which was used as an antioxidant in neuropathic pain and inflammatory pain models. Therefore, ROS are involved in central sensitization through NMDA receptor activation.

In the present study, pNR1 expression was decreased by which was inhibited the superoxide mediated by xanthine oxidase (XO), superoxide and NO (precursors of peroxynitrite), indicating that ROS are involved in the central sensitization mechanism associated with the NMDA receptor in the SCI model. Previous studies on the hippocampus and other cerebral regions have demonstrated that long-term potentiation (LTP) in synaptic efficacy may be produced postsynaptically by an alteration in patterns of presynaptic stimulation. It has been demonstrated that intense, recurrent and/or sustained noxious stimulation of C fibers leads to an increase in synaptic efficacy and wide dynamic range neuron excitability in the dorsal horn (25,26). Processes leading to central sensitization are associated with the processes underlying LTP and involve the engagement of NMDA receptors. Using electrophysiological and pharmacological approaches, NMDA receptors have been shown to have an important role in neuropathic pain (27,28). In the present study, a significant increase in pNR1 immunoreactivity was observed in the superficial laminae of dorsal horns from rats with neuropathic pain in an SCI model. This discrepancy may be accounted for by differences in the animal models used, time points or the methods used for the detection of pain behavior. Nociceptive behavior was assessed using the paw withdrawal threshold. However, Zou *et al* (14) demonstrated that only pNR1 in the spinal dorsal horn was enhanced following SCI, not NR1 itself. The sensitization of spinal dorsal horn neurons is expressed very rapidly and reversibly (14). However, the sensitization of spinal horn neurons induced by SCI, underlying chronic, pathological and painful states, lasts a long time, and may be associated with alterations in gene expression of NR1, and ultimately, morphological changes in NR1 expression.

In conclusion, superoxide produced by XO, and superoxide and NO as precursors of peroxynitrite, were demonstrated to be involved in the mediation of central sensitization associated with the phosphorylation of the NMDA receptor in an SCI model. These ROS contributed to the etiology and maintenance of mechanical allodynia. Therefore, the observations indicate that SOD, an ROS scavenger, may be used as a potential therapeutic agent to reduce CCP in patients following SCI.
